# Interleukin-10 rs1800896 polymorphism is associated with increased head and neck cancer risk but not associated with its clinical stages

**DOI:** 10.18632/oncotarget.16660

**Published:** 2017-03-29

**Authors:** Wei Huang, Juan Song, Xiao-Wei Jia, Yin-Xue Chen, Jia Shi, Xun Jiang

**Affiliations:** ^1^ Department of Stomatology, Zhuhai People's Hospital, Zhuhai Hospital Affiliated with Jinan University, Zhuhai 519099, China; ^2^ Department of Clinical Laboratory, Taihe Hospital, Hubei University of Medicine, Shiyan 442000, China; ^3^ Department of Stomatology, Guangzhou Hospital of Integrated Traditional and West Medicine, Guangzhou 510800, China

**Keywords:** interleukin-10, polymorphism, head and neck neoplasms, mouth neoplasms, oropharyngeal neoplasms

## Abstract

**Background:**

The association of interleukin-10 rs1800896 polymorphism with head and neck cancer risk and its clinical stages has been investigated by many published studies, but the results remain inconsistent. Therefore, we conducted this meta-analysis for further investigation.

**Results:**

Six case-control studies involving 1,781 head and neck cancer patients and 1,978 controls were yielded. The results indicated an association between rs1800896 polymorphism and increased head and neck risk [odds ratio (95%confidence interval) for G vs. A, GA vs. AA, GG vs. AA, GA+GG vs. AA, and GG vs. AA + GA were 1.63 (1.30–2.04), 3.17 (2.11–4.76), 1.63 (1.17–2.26), 1.73 (1.25–2.39), and 2.73 (1.82–4.09), respectively]. The subgroup analyses all obtained similar results with overall populations. The results of clinical stages yielded a non-significant association. No publication bias was detected.

**Materials and Methods:**

The PubMed and Chinese National Knowledge Infrastructure databases were searched up to December 27, 2016. Two authors independently selected studies, extracted and analyzed the data using the RevMan 5 software. Either a fixed effect or a random effect model was used to estimate pooled odds ratio and its 95% confidence intervals.

**Conclusions:**

We concluded that interleukin-10 rs1800896 polymorphism was significantly associated with head and neck cancer risk but not with the clinical stages thereof.

## INTRODUCTION

Head and neck cancer (HNC) majorly includes cancers starting from oral cavity, pharynx, and larynx. The patients would suffer problems in communication or swallowing after surgerical treatment due to the special position; hence, it is important to seek risk factors of the disease for preventing its onset. Factors such as lack of toothbrushing [1], tobacco smoking [2], periodontal diseases [3], betel quid chewing [4], alcohol drinking [5] and tooth loss [6] have been reported to be associated with increased risk of HNC. However, the susceptibility to HNC differs among persons exposed to the same environmental risk factors. That because genetic background is also responsible for the disease occurrence [7].

The interleukin-10 (IL-10) is a multifunctional immunosuppressant cytokine that is reported to be related to cancer onset and development [8]. It is located between 1q31 and 1q32 in human chromosome 1 [9] and the level of IL-10 gene expression can be strongly influenced by polymorphisms therein, such as the A-1082G (rs1800896) polymorphism which is in the promoter region [10]. In 2008, Vairaktaris et al. [11] conducted a case-control study and found that IL-10 rs1800896 polymorphism is strongly associated with increased risk for oral squamous cell carcinoma (OSCC) in Caucasians. However, the following relevant studies produced conflicting results. Yao et al. [12] obtained a significant association but Jeong et al. [13] yielded a non-significant association. Besides, the published two meta-analyses both consider nasopharyngeal cancer as HNC [14–15]. For meta-analysis is a useful tool to combine different results and may yield more precise results than individual genetic association studies [14–19], we further investigated the correlation between IL-10 rs1800896 polymorphism and risk of HNC by performing this meta-analysis. We also investigated the association between IL-10 rs1800896 polymorphism and HNC clinical stages.

## RESULTS

### Study characteristics

**Figure 1:**
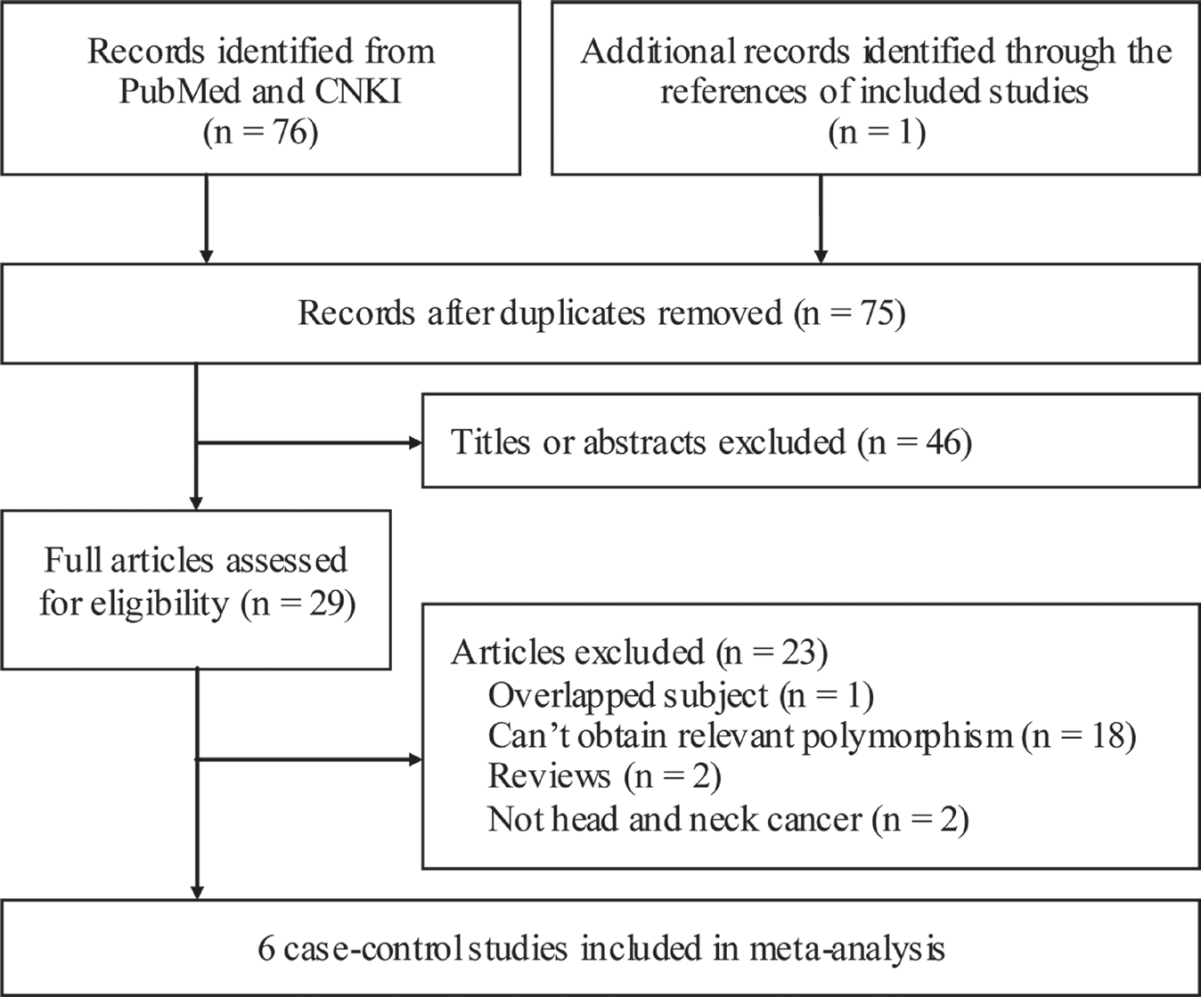
Flowchart of study section in the meta-analysis

The initial search identified 77 publications and finally 6 studies [11–13, 20–22] containing 1,781 HNC patients and 1,978 controls were included into this meta-analysis. The process of literature retrieval and selection are shown in Figure 1. Specifically, six studies were focused on IL-10 rs1800896 polymorphism and risk of HNC [11–13, 20–22], two studies including 290 HNC patients were focused on IL-10 rs1800896 polymorphism and clinical stages of HNC [11, 21]. One study were concerned about Caucasians [11] and the other five were about Asians. Three were focused on squamous cell carcinoma (SCC) only [11, 13, 21]. The characteristics and relevant data of the included studies are shown in Table 1.

**Table 1:** Main characteristics of all studies included in the meta-analysis

References	Country	Cancer	Gender (Male)*	Smoker*	Sample size*	G allele*	A allele*	Source of controls	Genotyping method	HWE
Vairaktaris 2008	Greece and Germany (Caucasian)	OSCC	114/105	134/88	144/141	100/60	188/222	Healthy volunteers	PCR-RFLP	No
Yao 2008	China (Asian)	Oral cancer (262 OSCC)	191/188	193/187	280/300	117/76	443/524	Healthy volunteers	PCR-RFLP	No
Jeong 2010	Korea (Asian)	HNSCC	252/339	NR	278/350	42/47	514/653	Hospital-based	PCR-TaqMan	Yes
Tsai 2014	China (Asian)	Oral cancer	599/727	595/667	788/956	315/212	1261/1700	Healthy volunteers	PCR	No
Zhou 2014	China (Asian)	LSCC	142/114	107/36	146/119	36/13	256/225	Healthy volunteers	PCR	Yes
Hsu 2015	China (Asian)	Oral cancer (148 OSCC)	125/77	116/38	145/112	16/16	274/208	Healthy volunteers	PCR-SSP	Yes
Clinical stages (All for patients)										
Vairaktaris 2008	Greece and Germany (Caucasian)	OSCC	114	134	144	52/38	108/90	NA	PCR-RFLP	NA
Zhou 2014	China (Asian)	LSCC	142	107	146	16/16	168/81	NA	PCR	NA

* case/control; OSCC, oral squamous cell carcinoma; HNSCC, head and neck squamous cell carcinoma; LSCC, laryngeal squamous cell carcinoma; NA, not available.

### IL-10 A-1082G polymorphism and HNC risk

**Figure 2:**
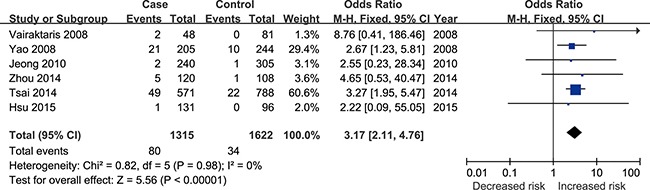
Forest plot of overall population of IL-10 rs1800896 polymorphism and risk of head and neck cancer (GG vs. AA model)

The combined results from the included six studies showed that IL-10 rs1800896 polymorphism is associated with increased risk of HNC under all the five genetic models: [G vs. A: odds ratio (OR) = 1.63, 95% confidence interval (CI) = 1.30–2.04 (Supplementary Figure 1); GG vs. AA: OR = 3.17, 95% CI = 2.11–4.76 (Figure 2); GA vs. AA: OR = 1.63, 95% CI = 1.17–2.26 (Supplementary Figure 2); GA+GG vs. AA: OR = 1.73, 95% CI = 1.25–2.39 (Supplementary Figure 3); and GG vs. AA + GA: OR = 2.75, 95% CI = 1.83–4.11 (Supplementary Figure 4)].

The source of controls of five studies was healthy volunteers [11–12, 20–22], all five genetic models showed significant association, which was also revealed in the results of Asians based on five studies [12–13, 20–22] and subgroup analysis of SCC type, mixed type and oral cancer under different genetic models. Table 2 shows the results of these analyses.

**Table 2:** Results of overall and subgroup meta-analysis

	No. of trials	Heterogeneity		Model	Meta-analysis	
		*p*	*I*^2^ (%)		OR (95% CI)	*p*
**G vs. A**	6	0.07	51	REM	1.63 (1.30–2.04)	< 0.01
Asian	5	0.05	58	REM	1.55 (1.18–2.03)	0.002
Healthy volunteers	5	0.16	40	FEM	1.77 (1.53–2.03)	< 0.01
SCC type	3	0.08	61	REM	1.70 (1.10–2.63)	0.02
Mixed type	3	0.08	61	REM	1.57 (1.42–2.16)	0.006
Oral cancer	4	0.13	47	REM	1.74 (1.50–2.01)	< 0.01
**GG vs. AA**	6	0.98	0	FEM	3.17 (2.11–4.76)	< 0.01
Asian	5	0.98	0	FEM	3.10 (2.05–4.67)	< 0.01
Healthy volunteers	5	0.94	0	FEM	3.19 (2.11–4.82)	< 0.01
SCC type	3	0.82	0	FEM	4.49 (1.10–18.27)	0.04
Mixed type	3	0.9	0	FEM	3.05 (2.00–4.67)	< 0.01
Oral cancer	4	0.88	0	FEM	3.13 (2.06–4.77)	< 0.01
**GA vs. AA**	6	0.009	67	REM	1.63 (1.17–2.26)	0.004
Asian	5	0.03	64	REM	1.46 (1.04–2.05)	0.03
Healthy volunteers	5	0.03	62	REM	1.78 (1.27–2.50)	< 0.01
SCC type	3	0.02	76	REM	1.85 (0.97–3.54)	0.06
Mixed type	3	0.03	71	REM	1.46 (0.93–2.30)	0.1
Oral cancer	4	0.02	71	REM	1.71 (1.15–2.54)	0.008
**GA + GG vs. AA**	6	0.007	69	REM	1.73 (1.25–2.39)	< 0.01
Asian	5	0.01	68	REM	1.56 (1.10–2.22)	0.01
Healthy volunteers	5	0.04	61	REM	1.92 (1.39–2.64)	< 0.01
SCC type	3	0.01	76	REM	1.94 (1.02–3.69)	0.04
Mixed type	3	0.02	73	REM	1.59 (1.02–2.47)	0.04
Oral cancer	4	0.02	70	REM	1.83 (1.26–2.66)	0.001
**GG vs. AA + GA**	6	0.99	0	FEM	2.75 (1.83–4.11)	< 0.01
Asian	5	0.99	0	FEM	2.70 (1.79–4.06)	< 0.01
Healthy volunteers	5	0.98	0	FEM	2.73 (1.81–4.12)	< 0.01
SCC type	3	0.93	0	FEM	3.75 (0.91–15.36)	0.07
Mixed type	3	0.93	0	FEM	2.66 (1.74–4.06)	< 0.01
Oral cancer	4	0.96	0	FEM	2.70 (1.78–4.10)	< 0.01

SCC, squamous cell carcinoma; REM, random-effect model; FEM, fixed effect model.

**Figure 3:**
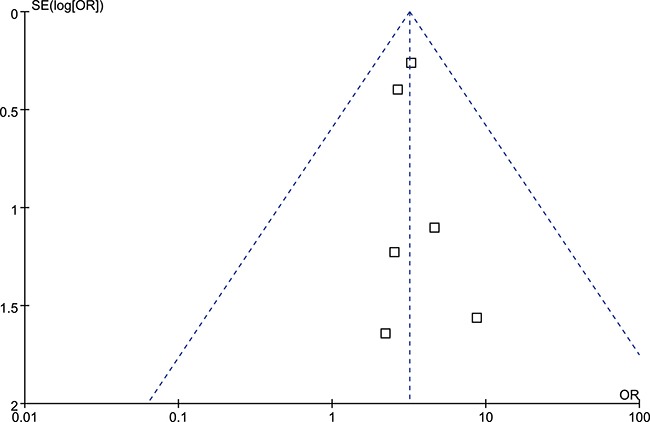
Funnel plot of overall population of IL-10 rs1800896 polymorphism and risk of head and neck cancer (GG vs. AA model)

The funnel plots of all five genetic models have well symmetry (Figure 3), indicated that there might be no obviously publication bias existed.

### IL-10 A-1082G polymorphism and HNC stages

**Figure 4:**
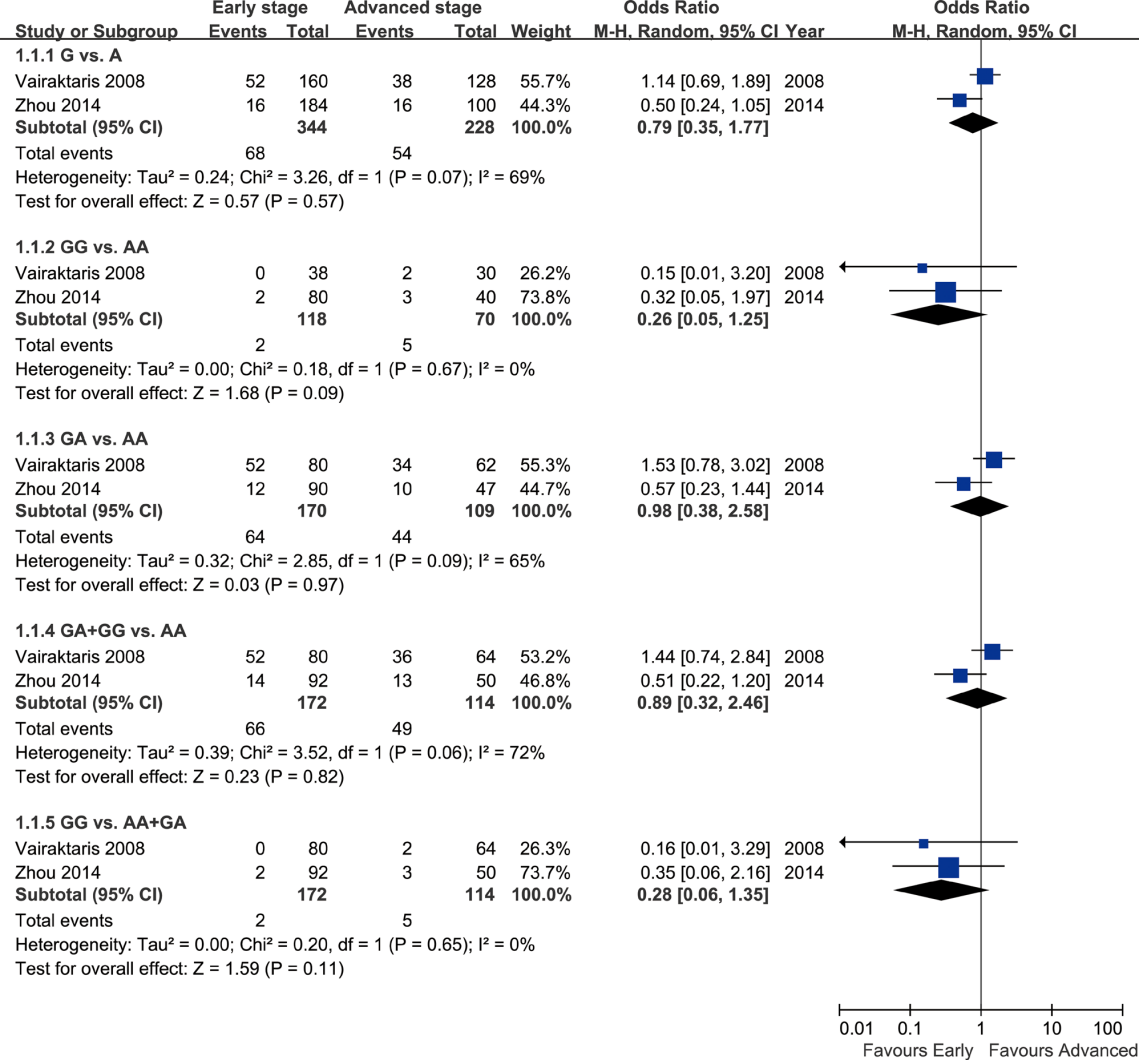
Forest plot of overall population of IL-10 rs1800896 polymorphism and clinical stages of head and neck cancer

The pooled results of two studies [11, 21] indicated that IL-10 rs1800896 polymorphism was not associated with clinical stages of HNC (Figure 4).

## DISCUSSION

Six case-control studies exploring the relationship between IL-10 rs1800896 polymorphism and risk of HNC were incorporated into the present meta-analysis. Of them, four studies reported a significant impact of IL-10 rs1800896 polymorphism on HNC risk [11–12, 20–21], but the other two showed apparent linkage there between [13, 22]. The results of our meta-analysis based on these six case-control studies indicated that IL-10 rs1800896 was related to increased susceptibility to HNC. The subgroup analyses of ethnicity, pathology types, source of controls, and cancer sites all revealed a significant association. Two studies reported no obvious effects of IL-10 rs1800896 on clinical stages of HNC [11, 21] and the pooled results of them also failed to identify a significant linkage.

HNC is a multifactorial disease [23–24], and genetic background is of great significance in its etiology through involvement in the disease onset and development [7]. With the development of molecular epidemiological technologies, more and more studies have been conducted to seek genetic polymorphisms related to HNC risk. IL-10 rs1800896 polymorphism G allele has been shown to cause higher IL-10 production [10], which may further lead to carcinogenesis. Some studies have illustrated that the IL-10 rs1800896 polymorphism was correlated with enhanced risk of nasopharyngeal carcinoma [25] and gastric cancer [26], diabetic nephropathy [27], and non-Hodgkin's lymphoma [28]. However, non significant correlation between the polymorphism and polycystic ovary syndrome [29], cervical cancer [30], periodontal diseases [31] has been revealed. This means that IL-10 is disease-specific and whether it has an influence on HNC risk remains a confusing due to the inconsistent results of published case-control studies.

By identifying these susceptible polymorphisms, population at high disease risk can be detected and prognosis of target disease can be predicted; it could be inferred from this meta-analysis that the population with rs1800896 polymorphism might have higher risk of developing HNC, and HNC patients at early stages with this mutation might be not likely to develop into advanced stages very quickly. Of course, this meta-analysis also indicated that IL-10 rs1800896 polymorphism could be used to identify and diagnose high risk population of HNC, but might be not valuable for judging the prognosis of HNC patients or the clinical stages.

Compared with the two published meta-analyses [14–15], our meta-analysis has some advantages. Nasopharyngeal cancer is not appropriate to be treated as HNC; however, these two meta-analyses [14–15] both included nasopharyngeal cancer. The meta-analysis by Niu et al. [14] included 5 case-control studies [11–13, 20, 22] for HNC and 4 for nasopharyngeal cancer, and it was very obvious that the study by Zhou et al. [21] in 2014 was missed. The meta-analysis by Li et al. [15] in 2016 only included 2 case-control studies [11–12] for HNC and more relevant studies were not take into consideration. Our meta-analysis, however, incorporated 6 case-control studies, and also investigated the impact of IL-10 rs1800896 polymorphism on HNC clinical stages.

Some limitations of our meta-analysis needed to be mentioned. The sample size was not large enough, especially in the analysis of clinical stages. As a result, the preciseness of true associations might be reduced, and subgroup analyses based on smoking status, gender, and ethnicity failed to be conducted due to lack of information. Second, the heterogeneity under some genetic models was significant, which also the case was found in the subgroup analyses (Table 2) although heterogeneity is a common phenomenon observed in the meta-analysis of genetic association studies [16–18, 30–40]. We also performed subgroup analyses to explore the origin of heterogeneity and found that it was not from clinical factors; in other words, the heterogeneity was just in the statistical level. Third, due to lack of adjusted data form original studies, our results were obtained based on unadjusted data. This might reduce the accuracy of final results by also involving other confounding factors such as smoking, gender, and alcoholism, the effects of the gene-gene or gene-environment interactions on HNC were not analyzed. Finally, due to the researcher's right of databases and language ability, only two comprehensive databases of PubMed and CNKI were searched for retrieval of eligible studies, and the searching language was limited to English and Chinese.

In summary, our meta-analysis indicated that IL-10 rs1800896 polymorphism might contribute to enhanced HNC susceptibility but had no apparent relationship with clinical stages of HNC. However, due to the limitations of our meta-analysis, more larger and well-designed prospective studies are needed to be performed to further confirm our findings.

## MATERIALS AND METHODS

### Eligible criteria

We included studies meeting all the following criteria: (1) the patients were diagnosed as HNC by pathological methods and the controls were healthy volunteers or HNC-free patients; (2) the exposure was IL-10 rs1800896 polymorphism and the study was a case-control or cohort study design; (3) the associations between IL-10 rs1800896 polymorphism and risk and/or clinical stages of HNC were explored; (4) complete information of necessary genotypes or other sufficient data to calculate them were reported. If the same institute published two or more publications, we treated them as independently ones and chose the more comprehensive one.

### Search strategy

A comprehensive literature search was performed in PubMed and CNKI (Chinese National Knowledge Infrastructure) up to December 27, 2016. The following search term was used: (interleukin-10 OR IL-10) AND (polymorphism OR mutation OR variant OR variation) AND (carcinoma OR cancer OR tumor OR neoplasm) AND (head and neck OR oral OR pharyngeal OR oropharynx OR laryngeal OR laryngopharyngeal OR mouth OR tongue). Moreover, all listed references of included studies and recently reviews were also retrieved for potential articles. No language restriction was applied. Supplementary Table 1 presents the search strategy of PubMed.

### Data extraction

Study selection and data extraction were performed by two authors independently and any possible discrepancy was resolved by discussion. The following data was extracted from each included study: surname of first author, year of publication, study design, country and ethnicity of study population, demographics, cancer sites, histopathologic types, smoking status, clinical stages, number of cases and controls, genotype distribution, source of controls, genotyping method, and Hardy-Weinberg equilibrium (HWE) for controls. The HWE was calculated if it was not reported and the level of *P* < 0.05 was considered as not conforming to HWE.

### Data analysis

ORs and their 95% CIs were calculated to estimate the relationship under five genetic models of G vs. A, GG vs. AA, GA vs. AA, GA + GG vs. AA, and GG vs. AA + GA. Heterogeneity was assessed using the Cochran's *Q* statistic and *I*^2^ statistic [41–42] firstly, with *P* ≥ 0.1 and *I*^2^ < 50% indicating acceptable heterogeneity, in which case the fixed effect model was used; otherwise, the random-effects model was used. The clinical stages were evaluated using the early stage (stages I and II) vs. advanced stage (stages III and IV). If the number of included studies was available, we conducted subgroup analyses based on the ethnicity, pathology types, and source of controls, cancer sites and smoking status. Publication bias was assessed by funnel plot [43]. All analyses were performed using the Review Manager (RevMan) 5.3 software [3, 17].

## SUPPLEMENTARY MATERIALS TABLES AND FIGURES


