# Supplementary material for: Interleukin-10 rs1800896 polymorphism is associated with increased head and neck cancer risk but not associated with its clinical stages
Source: Oncotarget. 2017 Mar 29;8(23):37217–24. doi: 10.18632/oncotarget.16660 (PMC5514904; doi:10.18632/oncotarget.16660)
Supplement: Supplementary file 1 [file oncotarget-08-37217-s001.pdf]

Interleukin-10 rs1800896 polymorphism is associated with increased head and neck cancer risk but not associated with its clinical stages

Supplementary Materials

Supplementary Table 1: The search strategy of PubMed

No.	Query Results	Results
#20	Search ((((IL-10)) AND (((((polymorphism)) OR (mutation)) OR (variant)) OR (variation))) AND (((((carcinoma)) OR (cancer)) OR (tumor)) OR (neoplasm))) AND ((((((((head and neck)) OR (oral)) OR (pharyngeal)) OR (oropharynx)) OR (laryngeal)) OR (laryngopharyngeal)) OR (mouth)) OR (tongue))	62
#19	Search ((((((((head and neck)) OR (oral)) OR (pharyngeal)) OR (oropharynx)) OR (laryngeal)) OR (laryngopharyngeal)) OR (mouth)) OR (tongue)	1179773
#18	Search “tongue”[MeSH Terms] OR “tongue”[All Fields]	47911
#17	Search “mouth”[MeSH Terms] OR “mouth”[All Fields]	350250
#16	Search “hypopharynx”[MeSH Terms] OR “hypopharynx”[All Fields] OR “laryngopharyngeal”[All Fields]	6804
#15	Search “larynx”[MeSH Terms] OR “larynx”[All Fields] OR “laryngeal”[All Fields]	87310
#14	Search “oropharynx”[MeSH Terms] OR “oropharynx”[All Fields]	19031
#13	Search “pharynx”[MeSH Terms] OR “pharynx”[All Fields] OR “pharyngeal”[All Fields]	66492
#12	Search “mouth”[MeSH Terms] OR “mouth”[All Fields] OR “oral”[All Fields]	949894
#11	Search “Head Neck”[Journal] OR (“head”[All Fields] AND “and”[All Fields] AND “neck”[All Fields]) OR “head and neck”[All Fields]	131684
#10	Search ((((carcinoma)) OR (cancer)) OR (tumor)) OR (neoplasm)	3836058
#9	Search “tumour”[All Fields] OR “neoplasms”[MeSH Terms] OR “neoplasms”[All Fields] OR “tumor”[All Fields]	2946045
#8	Search “neoplasms”[MeSH Terms] OR “neoplasms”[All Fields] OR “cancer”[All Fields]	3443811
#7	Search “carcinoma”[MeSH Terms] OR “carcinoma”[All Fields]	819451
#6	Search (((“polymorphism, genetic”[MeSH Terms] OR (“polymorphism”[All Fields] AND “genetic”[All Fields]) OR “genetic polymorphism”[All Fields] OR “polymorphism”[All Fields]) OR (“mutation”[MeSH Terms] OR “mutation”[All Fields])) OR variant[All Fields]) OR variation[All Fields]	1499075
#5	Search variation[All Fields]	481305
#4	Search variant[All Fields]	149320
#3	Search “mutation”[MeSH Terms] OR “mutation”[All Fields]	783219
#2	Search “polymorphism, genetic”[MeSH Terms] OR (“polymorphism”[All Fields] AND “genetic”[All Fields]) OR “genetic polymorphism”[All Fields] OR “polymorphism”[All Fields]	277192
#1	Search “interleukin-10”[MeSH Terms] OR “interleukin-10”[All Fields] OR “il 10”[All Fields]	50934

Study or Subgroup	Case Events	Case Total	Control Events	Control Total	Weight	Odds Ratio M-H, Random, 95% CI	Year
Yao 2008	117	560	76	600	21.1%	1.82 [1.33, 2.50]	2008
Vairaktaris 2008	100	288	60	282	18.0%	1.97 [1.35, 2.86]	2008
Jeong 2010	42	556	47	700	15.5%	1.14 [0.74, 1.75]	2010
Zhou 2014	36	292	13	238	8.8%	2.43 [1.26, 4.70]	2014
Tsai 2014	315	1756	212	1912	28.8%	1.75 [1.45, 2.11]	2014
Hsu 2015	16	290	16	224	7.8%	0.76 [0.37, 1.55]	2015
**Total (95% CI)**		**3742**		**3956**	**100.0%**	**1.63 [1.30, 2.04]**	
Total events	626		424				

Heterogeneity: Tau² = 0.04; Chi² = 10.23, df = 5 (P = 0.07); I² = 51%
Test for overall effect: Z = 4.24 (P < 0.0001)

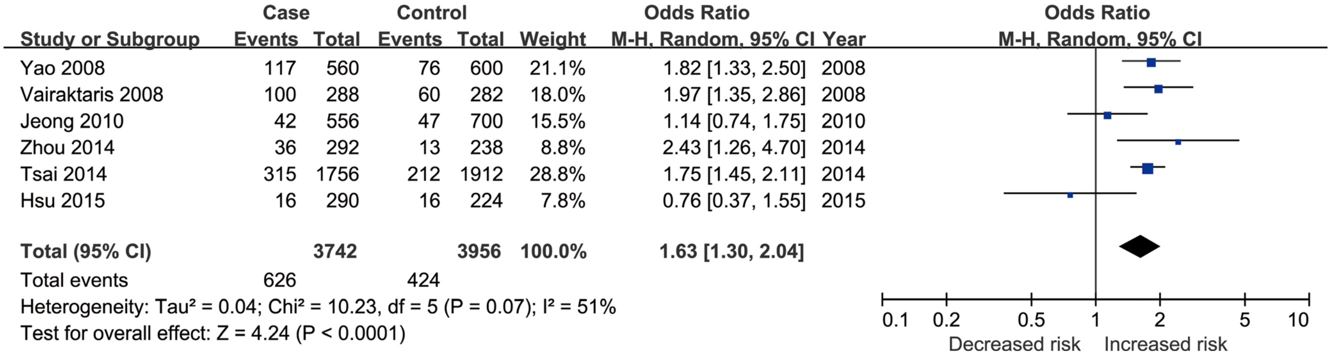

Supplementary Figure 1: Forest plot of overall population of IL-10 rs1800896 polymorphism and risk of head and neck cancer (G vs. A model).

Study or Subgroup	Case Events	Case Total	Control Events	Control Total	Weight	Odds Ratio M-H, Random, 95% CI	Year
Yao 2008	75	259	56	290	19.4%	1.70 [1.15, 2.53]	2008
Vairaktaris 2008	96	142	60	141	17.0%	2.82 [1.74, 4.57]	2008
Jeong 2010	38	276	45	349	17.6%	1.08 [0.68, 1.72]	2010
Tsai 2014	217	739	168	934	23.9%	1.90 [1.51, 2.39]	2014
Zhou 2014	26	141	11	118	11.2%	2.20 [1.04, 4.67]	2014
Hsu 2015	14	144	16	112	11.0%	0.65 [0.30, 1.39]	2015
**Total (95% CI)**		**1701**		**1944**	**100.0%**	**1.63 [1.17, 2.26]**	
Total events	466		356				

Heterogeneity: Tau² = 0.10; Chi² = 15.25, df = 5 (P = 0.009); I² = 67%
Test for overall effect: Z = 2.91 (P = 0.004)

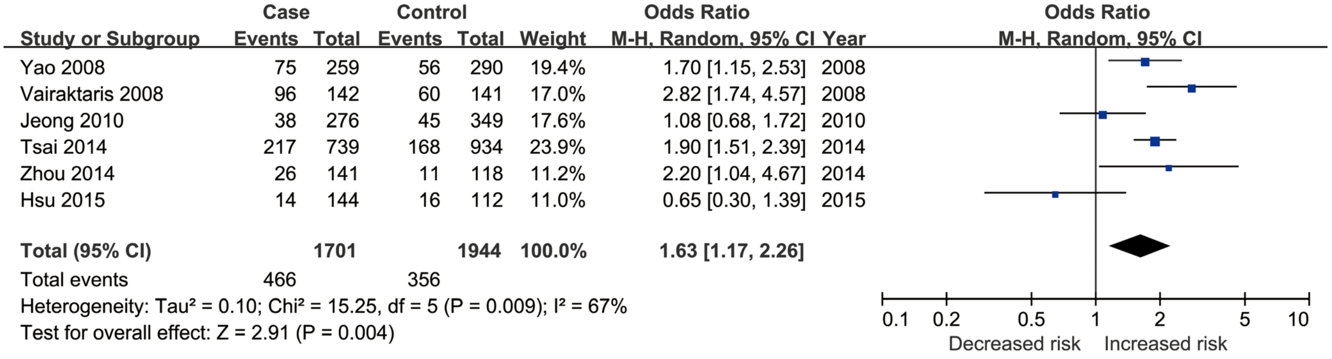

Supplementary Figure 2: Forest plot of overall population of IL-10 rs1800896 polymorphism and risk of head and neck cancer (GA vs. AA model).

Study or Subgroup	Case Events	Case Total	Control Events	Control Total	Weight	Odds Ratio M-H, Random, 95% CI	Year
Vairaktaris 2008	98	144	60	141	16.7%	2.88 [1.77, 4.67]	2008
Yao 2008	96	280	66	300	19.8%	1.85 [1.28, 2.67]	2008
Jeong 2010	40	278	46	350	17.4%	1.11 [0.70, 1.75]	2010
Tsai 2014	266	788	190	956	23.8%	2.05 [1.65, 2.55]	2014
Zhou 2014	31	146	12	119	11.5%	2.40 [1.17, 4.92]	2014
Hsu 2015	15	145	16	112	10.9%	0.69 [0.33, 1.47]	2015
**Total (95% CI)**		**1781**		**1978**	**100.0%**	**1.73 [1.25, 2.39]**	
Total events	546		390				

Heterogeneity: Tau² = 0.10; Chi² = 15.95, df = 5 (P = 0.007); I² = 69%
Test for overall effect: Z = 3.33 (P = 0.0009)

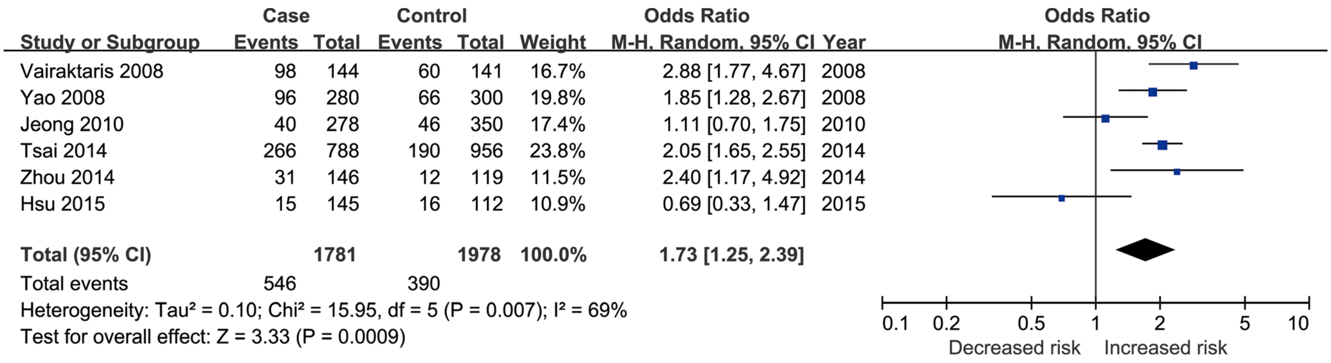

Supplementary Figure 3: Forest plot of overall population of IL-10 rs1800896 polymorphism and risk of head and neck cancer (GA+GG vs. AA model).

Study or Subgroup	Case Events	Case Total	Control Events	Control Total	Weight	Odds Ratio M-H, Fixed, 95% CI	Year
Vairaktaris 2008	2	144	0	141	1.6%	4.96 [0.24, 104.34]	2008
Yao 2008	21	280	10	300	29.2%	2.35 [1.09, 5.09]	2008
Jeong 2010	2	278	1	350	2.9%	2.53 [0.23, 28.04]	2010
Zhou 2014	5	146	1	119	3.5%	4.18 [0.48, 36.32]	2014
Tsai 2014	49	788	22	956	61.0%	2.81 [1.69, 4.70]	2014
Hsu 2015	1	145	0	112	1.8%	2.34 [0.09, 57.88]	2015
**Total (95% CI)**		**1781**		**1978**	**100.0%**	**2.75 [1.83, 4.11]**	
Total events	80		34				

Heterogeneity: Chi² = 0.47, df = 5 (P = 0.99); I² = 0%
Test for overall effect: Z = 4.89 (P < 0.00001)

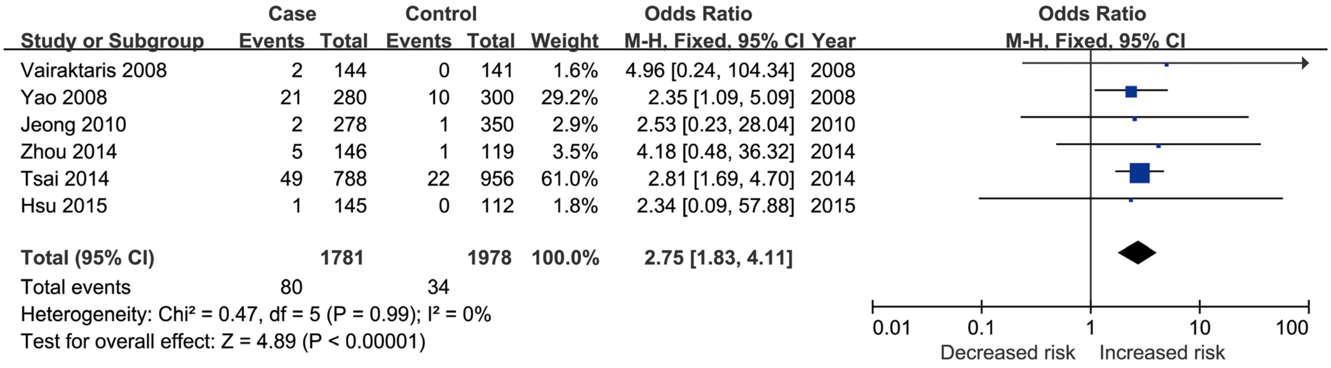

Supplementary Figure 4: Forest plot of overall population of IL-10 rs1800896 polymorphism and risk of head and neck cancer (GG vs. AA + GA model).
